# Population pharmacokinetics of standard-dose meropenem in critically ill patients on continuous renal replacement therapy: a prospective observational trial

**DOI:** 10.1007/s43440-020-00104-3

**Published:** 2020-04-16

**Authors:** Dariusz Onichimowski, Anita Będźkowska, Hubert Ziółkowski, Jerzy Jaroszewski, Michał Borys, Mirosław Czuczwar, Paweł Wiczling

**Affiliations:** 1 Department of Anesthesiology and Intensive Care, Faculty of Medicine, University of Warmia and Mazury, Ul. Żołnierska 18, 10-900 Olsztyn, Poland; 2 Department of Biopharmaceutics and Pharmacodynamics, Medical University of Gdańsk, Gdańsk, Poland; 3 Department of Pharmacology and Toxicology, Faculty of Veterinary Medicine, University of Warmia and Mazury, Olsztyn, Poland; 4 2nd Department of Anaesthesiology and Intensive Therapy, Medical University of Lublin, Lublin, Poland

**Keywords:** Sepsis, Meropenem, Pharmacokinetic modelling, Critically ill, Renal replacement therapy

## Abstract

**Background:**

The primary objective of this study was to develop a population pharmacokinetic model of meropenem, based on the population of critically ill adult patients undergoing CRRT. The secondary one was to examine the relationship between patient characteristics (covariates) and individual PK parameters. Finally, we aimed to perform Monte Carlo simulations to assess the probability of target attainment (PTA) of %*T* > MIC considering the uncertainty of PK parameters.

**Materials and methods:**

The study population included 19 adult critically ill patients on CRRT, receiving 1 g of meropenem in 1-h infusions every 8 h. Blood samples were collected prior to (time zero) and 15, 30, 45, 60, 75, 90, 120, 180, 240 and 480 min after the start of meropenem administration. Population nonlinear mixed-effects modeling was conducted using NONMEM software, Fortran, and Wings for NONMEM.

**Results:**

A two-compartment model was used to describe the available data. Typical values of the central and peripheral volume of distribution, and the CRRT and inter-compartmental clearance for a theoretical patient with 24.6 g/l albumin concertation were *V*_1_ = 27.9 l, *V*_2_ = 33.7 l, *Cl*_CRRT_ = 15.1 l/h, and *Q* = 21.1 l/h. A significant covariate relationship between *V*_1_ and albumin concentration was observed in the data that was described by a power relationship with − 2.87 exponent. Subsequently performed Monte Carlo simulations of the model allowed us to assess the impact of albumin concentration on PTA. The 40%*T* > 2 mg/l target was reached in more than 90% of subjects after 1-h infusion of 1000 mg q8h and steady-state conditions. The more stringent 100%*T* > 2 mg/l target requires higher doses and/or longer infusion durations that depend on the albumin concentration.

**Conclusions:**

The population PK model was successfully developed to describe the time course of meropenem concentrations. The hypoalbuminemia was found to be associated with higher PTA in the CRRT patients after multiple short-term infusions.

**Electronic supplementary material:**

The online version of this article (10.1007/s43440-020-00104-3) contains supplementary material, which is available to authorized users.

## Introduction

Sepsis-associated morbidity and mortality remain very high despite significant advances in the quality of intensive care worldwide. The recent Surviving Sepsis Campaign guidelines provide multiple recommendations on the treatment of sepsis, yet meaningful data is still lacking, including both understanding the fundamental mechanisms of the pathophysiology of sepsis, as well as substantial clinical issues [1]. It is beyond doubt, that a better understanding of sepsis-induced pathophysiological changes, as well as more personalized approach are mandatory to improve outcomes, especially in patients presenting with multiorgan failure in the course of multidrug-resistant (MDR) bacterial infections.

One of the most urgently needed areas of research includes the pharmacokinetic and pharmacodynamic (PK/PD) optimization of antimicrobials because sepsis-induced pathophysiological changes significantly influence the ability of most antibiotics to reach therapeutic concentrations [2]. Patient factors associated with the inability of antibiotics to reach therapeutic concentrations in critically ill include increased epithelial leakage, low plasma protein levels, organ dysfunction, as well as fluctuating fluid balance. Furthermore, the broad implementation of extracorporeal techniques in the intensive care unit (ICU), such as continuous renal replacement therapy (CRRT), contributes to factors that need to be considered for potential effects on antibiotic dosing in critically ill patients which might help in overcoming the increasing bacterial resistance, burdened with significant mortality [3].

Carbapenems belong to the most widely utilized antimicrobials in treating life-threatening infections due to MDR Gram-negative bacteria in the ICUs worldwide. Their pharmacodynamic target for a maximal bactericidal activity depends on the time during which the free drug plasma concentration is maintained above the minimum inhibitory concentration (MIC). The results of experimental research demonstrated that the required time over MIC (%*T* > MIC) for carbapenems is about 40% of the dosing interval for the majority of the indications [4]. The more recent clinical studies, performed in the critically ill patients with sepsis or septic shock, challenged the beforementioned PK/PD goal and advocated the maintenance of trough level above the MIC for the entire dosing interval [5]. Furthermore, maximum efficacy may be anticipated by the minimum plasma concentration (*C*_min_) targeted to values at least four times the MIC value (*C*_min_ > 4 × MIC) or even as high as *C*_min_ > 5 × MIC [6, 7]. It should be noted, that achieving and maintaining the beforementioned PK/PD targets for carbapenems may be extremely difficult in the critically ill patients, considering the sepsis-induced pathophysiological alterations. It should be noted that the impact of the treatment modalities used in the ICU setting, such as mechanical ventilation, vasopressor support, fluid resuscitation, and extracorporeal therapies should also be taken into account [8].

Acute kidney injury (AKI) is diagnosed in nearly half of the population of critically ill patients with sepsis or septic shock and it is associated with an increase of in-hospital mortality [9, 10]. Authors of a recent trial confirmed the abovementioned estimates and demonstrated that renal replacement therapy (RRT) was required in about one-third of the studied ICU population [11]. It is noteworthy that continuous RRT modalities are recommended for hemodynamically unstable critically ill patients, however, to date there is no clear evidence on the impact of the choice of modality on outcome in this challenging patient population. The clinical data is also lacking on the possible influence of CRRT on the pharmacokinetics of antibiotics used to treat life-threatening infections in the ICU. According to the results of the RENAL trial, 25% of ICU patients receiving CRRT failed to achieve therapeutic concentrations of antibiotics regardless of the dialysis dose [12].

The objectives of this study were: (1) to develop population pharmacokinetic model of meropenem based on the population of adult patients receiving CRRT in an intensive care unit, (2) to examine the relationship between patient characteristics (covariates) and individual PK parameters to explain part of the inter-individual variability in PK parameters and (3) to perform Monte Carlo simulations to assess the probability of target attainment (PTA) for different dosing regimens. The following PK/pharmacodynamic (PD) targets were evaluated: the percentage of the dosing interval that the free drug concentration is maintained above the MIC of 40% and 100%.

## Materials and methods

### Patients and study design

This was a prospective, observational cohort study investigating the pharmacokinetics of meropenem in adult patients admitted to a tertiary medical/surgical ICU in Olsztyn, Poland. Ethical approval was obtained from the Bioethical Committee that belongs to the district Medical Chamber in Olsztyn. The following inclusion criteria for the study were used: age 18–80 years, both medical and surgical origin of ICU admission, treatment with licensed doses of meropenem (1 g every 8 h), and clinical indications for CRRT due to AKI, determined in accordance with the criteria adopted by Kidney Disease Improving Global Outcomes (KDIGO) recommendations [13]. Patients were excluded from the study if they were diagnosed with HIV infection or terminal cancer, displayed intolerance or allergy to beta-lactam antibiotics in the past, had a high probability of bacterial infection with meropenem resistant strains, and received meropenem up to 3 months before being screened.

Each patient received standard dosing of meropenem (Meronem^®^, AstraZeneca, Zug, Switzerland)—short term infusion of 1 g over 1 h every 8 h. Meropenem was diluted in 500 ml of physiologic saline (Fresenius Kabi Poland) and was administered during 1 h with the use of high-precision infusion pump (Fresenius Vial—Le Grand Chemin, Brezins, France). Arterial blood samples (2 ml) for PK analysis were collected into heparinized test tubes prior to (time zero) and 15, 30, 45, 60, 75, 90, 120, 180, 240 and 480 min after the start of meropenem administration.

### Continuous renal replacement therapy

Patients received CRRT with the use of Multifiltrate dialysis machine (Fresenius Medical Care, Germany) because of AKI and/or fluid overload. The vascular access for the CRRT procedure was obtained through either femoral or internal jugular vein. The CRRT treatment modality (continuous veno-venous hemofiltration; CVVH or continuous veno-venous hemodialysis; CVVHD) was chosen by the leading physician. The AV 1000 polisulfon membrane with the effective surface area of 1.8 m^2^ (Fresenius Medical Care, Germany) was used in both groups. The choice of anticoagulation method depended on the CRRT modality and systemic heparin was used for CVVH and regional citrate anticoagulation was used for CVVHD. In patients treated with CVVH the blood flow was set between 200 and 240 ml/min (mean ± SD 214.44 ± 18.10), however, during CVVHD flow was established at 110–160 ml/min (mean ± SD 136 ± 15.78). The flow of the dialysate during CVVH and substitute flow in the course of CVVHD were set at 35 ml/kg/h with a round up to 50 ml/h (mean ± SD 2753 ± 340.38). During CVVH event the substitute flow was set as pre and post-dilution in 1:2 ratio. The pure ultrafiltration (patient subtractions) rates varied from 0.00 to 0.35 (mean ± SD 125.26 ± 90.76).

### Pharmacokinetic assay

Blood samples (2 ml) were collected into heparin covered test-tubes from the arterial lines of the CRRT circuit just before (time 0) and 15, 30, 45, 60, 75, 90, 120, 180, 240, 480 min. after meropenem administration. Subsequently, test tubes were stored in cold bath (1 h maximal time) and centrifuge for 10 min with 3000×*g* to remove red blood cells. Finally, blood plasma was frozen at – 80 °C for further pharmacokinetic assay.

The analysis was provided by high-performance liquid chromatograph Agilent 1100 series (Agilent Technologies, Waldbronn, Germany) equipped with: double pump G1311A, degasser G1379A, auto sampler G1313A, column thermostat G1316A, and diode detector working in UV light (300 nm). The chromatographic separation was performed using Waters Atlantis T3 column (3.0/150 mm, 3.5 μm) working at 35 °C in gradient elution (flow rate: 0.35 ml/min): mobile phase A (87.5% 0,01 M potassium phosphate monobasic, 10% acetonitrile, 2.5% methanol, pH 3.5 using 100% glacial acetic acid); mobile phase B (15% 0.01 M potassium phosphate monobasic, 85% acetonitrile, pH 3.5 using 100% glacial acetic acid).

For extraction procedure 250 μl of the plasma was defrosted in the room temperature. Next to achieve proteins denaturation, 250 μl of the acetonitrile was added to the samples. The solution was mixed in a vortex for 30 s, 3000 RPMs, and followed by centrifugation (4000×*g*, 10 min, 4 °C). The supernatant was transferred to clean tubes and 1500 μl of 1,2-dichloroethane was added. Next the samples were mixed in a vortex for 60 s, 3000 RPMs and centrifuged (4000×*g*, 10 min., 4 °C). The 150 μl of the water phase was transferred to a clean tube and centrifuged again (4000×*g*, 10 min., 4 °C) to eliminate solid contaminations. Next, 150 μl of solution was transferred to Total Recovery vials and 5 μl of the sample was injected for chromatographic analysis (15 min). Samples, that were used to establish the calibration curve were prepared analogically to the tested samples. The applied analytical method was fully validated in our laboratory, according to the United States Food and Drug Administration (FDA) and European Medicines Agency (EMA) bioanalytical method validation requirements (EMA, 2011; FDA, 2013).

To validate the procedure, the following were assessed: limit of detection (LOD), the lower limit of quantitation (LLOQ), linearity, accuracy, intra- and inter-day precision, specificity, stability, recovery, carry-over, and matrix effect. The LOD was 0.06 μg/ml ± 0.01 (signal:noise ratio ≥ 3:1); and the LLOQ, 0.1 μg/ml ± 0.03 (signal:noise ratio ≥ 6:1).

To prepare the calibration curve for assessing linearity, plasma that was free of meropenem was separated from blood obtained from clinically healthy subjects. Then, a set of samples was prepared for constructing 10-point calibration curves (0.1, 0.5, 1.0, 2.5, 5.0, 10.0, 20.0, 40.0, 75.0, 100.0 µg/ml). Before constructing the curves, a blank sample without any analytes was measured. Then, each set of samples was measured in two replicates. The entire process was repeated four times at 1-day intervals. Linearity was high, as shown by *r*^2^ values of 0.99 for all curves. For quality control, four concentrations were used: low quality control (LQC 0.5 μg/ml), intermediate quality control (IQC 5.0 μg/ml), medium quality control (MQC − 20.0 μg/ml), and high-quality control (HQC 75.0 μg/ml). Accuracy was 2.92–8.66%; and precision, 2.69–8.99%. To determine the specificity of the method, six samples of meropenem-free plasma were used. Specificity was confirmed by a lack of significant peaks at the retention time of meropenem. Drug stability was confirmed in three ways. First, it was stored in an autosampler at 17 °C for 72 h, which resulted in an increase or decrease in the concentration of 2.84% ± 3.69. Second, it was subjected to a freeze–thaw cycle over 12 days, and its concentration decreased by 2% ± 0.77. Third, it was prepared as a working standard and stored in a refrigerator at 4 °C for 7 days, after which the increase/decrease was 4.58% ± 4.99. The total recovery of meropenem was 83.4% ± 10.23. There was no carry-over of the drug. To check for a potential matrix effect, the signals were compared after analyzing samples with the same concentrations of meropenem dissolved in the matrix before and after the extraction procedure, and the signal increase/decrease was only 3.76% ± 2.78.

### Population pharmacokinetic analysis

#### Population PK methods

The population PK modeling was performed with NONMEM software (version 7.3, Icon Development Solutions, Ellicott City, Maryland,* USA*), Fortran compiler (version 4.6.0) and Wings for NONMEM (version 741, https://wfn.sourceforge.net). The FOCE with interaction method using ADVAN3, TRANS4 routine was employed throughout the model-building procedure. Matlab^®^ (version 7.0, The MathWorks, Inc., Massachusetts,* USA*) was used to process and visualize data generated by NONMEM.

Models obtained in this work were assessed and compared using the minimum value of the NONMEM objective function (OFV), typical goodness-of-fit (GOF) diagnostic plots, and the evaluation of the precision of pharmacokinetic parameter and variability estimates.

#### Pharmacokinetic model

Based on literature a two-compartment model was used to describe plasma meropenem concentrations:

1 $$ V_{1} \frac{{{\text{d}}C_{1} }}{{{\text{d}}t}} = - Q \times C_{1} + Q \times C_{2} - {\text{Cl}}_{{{\text{CRRT}}}} \times C_{1} $$

2 $$ V_{2} \frac{{{\text{d}}C_{2} }}{{{\text{d}}t}} = Q \times C_{1} - Q \times C_{2} , $$

where *t* denotes time, *C*_1_ and *C*_2_ denote meropenem concentration in central and peripheral compartment, Cl_CRRT_ and *Q* denote the systemic (due to CRRT) and inter-compartmental clearance; and *V*_1_ and *V*_2_ denote the volume of distribution of central and peripheral compartment.

Inter-individual variability (IIV) for PK parameters was modeled in terms of *η* (the difference in parameter value between the individual and typical patient on a log scale):

3 $$ P_{i} = \theta \times \exp \left( {\eta_{P,i} } \right), $$

where *P*_*i*_ is the individual PK parameter, *θ* is the typical value of this PK parameter in the population, and $\eta_{P,i}$ is a random effect for that PK parameter associated with between-individual variability. The *η* was assumed to have normal distributions with mean 0 and variances $\omega_{P}^{2}$.

The residual error for observations was modeled using an additive and proportional error model:

4 $$ C_{{{\text{obs}}}} = C_{1} + C_{1} \times \varepsilon_{{{\text{prop}}}} + \varepsilon_{{{\text{add}}}} , $$

where *C*_obs_ and *C*_1_ are observed and predicted (Eq. 1) meropenem concentrations and *ε*_add_ and *ε*_prop_ represent the additive and proportional component of residual variability of meropenem concentrations. It was assumed that *ε*_add_ and *ε*_prop_ and is normally distributed with the mean 0 and variances $\sigma_{{{\text{add}}}}^{2}$ and $\sigma_{{{\text{prop}}}}^{2}$.

#### Covariance analysis

After the appropriate base (structural) model was established, 17 covariates, including age, body weight, day of antibiotic therapy, presence or absence of sepsis according to Surviving Sepsis Compaigne, gender, place on the APACHE II and SOFA scale, serum albumin level, creatinine concentration, eGFR_MDRD_ (estimated with the MDRD equation), eGFR_CG_ (estimated with the Cockroft-Gault equation), diuresis and parameters of CRRT: type of anticoagulation, day of filter usage, blood flow, dialysate/substitute flow, UF net were assessed as a potential covariates. For this purpose, the estimates of *η*_*P*_ values were plotted against covariates to assess relationship. If a trend between a covariate and *η*_*P*_ values of the PK parameter was found it was considered in the base model. A linear and power relationships were tested according to the following equations:

5 $$ P_{i} = \theta_{P} \times \left( {1 + \theta_{{{\text{beta}}P}} \times \left( {{\text{COV}}_{i} - \overline{{{\text{COV}}}} } \right)} \right) \times \exp \left( {\eta_{P,i} } \right) $$

6 $$ P_{i} = \theta_{P} \times ({\text{COV}}_{i} /\overline{{\text{COV)}}}^{{\theta_{{{\text{beta}},P}} }} \times \exp \left( {\eta_{P,i} } \right), $$

where $\overline{{{\text{COV}}}}$ is a median of a covariate and $\theta_{{{\text{beta}},P}}$ is the regression or power coefficient. Categorical covariates were included in the model based on indicator variables:

7 $$ P_{i} = \theta_{P} \times \left( {1 + \theta_{{{\text{beta}}P}} \times {\text{IND}}_{i} } \right) \times \exp \left( {\eta_{P,i} } \right), $$

where IND_*i*_ is an indicator variable that has a value of 1 or 0.

Covariates were kept in the model if there were biologically plausible and their inclusion into the model led to a significant difference in OFV. The difference in the minimum of the NONMEM OFV obtained for the two nested models (likelihood ratio) is approximately *χ*^2^ distributed. The difference in OFV between models of 3.84 for 1 degree of freedom was considered to be statistically significant at *p* < 0.05 for the covariate to be included in the base model. This process was repeated until all significant covariates were added. Then backward elimination was performed by removing one covariate at a time. The least important covariate was dropped from the model according to the OFV unless that difference in OFV was larger than 7.9 (corresponding to *p* < 0.005). The final model was established when no more covariates could be excluded from the model.

#### Model evaluation

The model performance was assessed using visual predictive checks (VPC). For VPC the predicted PK profiles for 1000 virtual datasets were generated from the final parameters and variances estimates. The observed and predicted concentrations were binned across time. The predicted median along with 5th and 95th percentile of the simulated concentrations was plotted against time with the median and 5th and 95th percentiles of the observed concentrations. When the percentile from the experimental data falls outside the 90% confidence interval derived from predictions, it indicates model misspecification. A nonparametric bootstrap analysis was used to evaluate the uncertainty of model parameters. The data for individual patients were randomly sampled with replacement from original dataset to form 1000 new data sets. Each data contained the same number of patients as original dataset. In the next step, each dataset was fitted to the final model. The model parameters from bootstrap samples were summarized as a median and 90% (5th–95th percentile) confidence intervals.

#### Dosing simulations

Monte Carlo dosing simulations were performed using NONMEM for short-term infusion (0.5, 1 and 3 h) of 1 g of meropenem every 8 h (q8h) and every 12 h (g12h) for patients with three different albumin concentrations (15.6, 24.6 and 31.8 g/l). They correspond to the lowest, median, highest observed values. Each simulation generated steady-state concentration–time profiles for 1000 subjects using the final estimated population PK parameters. From these data the %*T* > MIC was calculated for each subject and then PTA was obtained by counting subjects who achieved 40% or 100% *T* > MIC for MIC values ranging from 0.064 to 64 mg/l and for albumin concentrations spanning the range of values observed in this study. The linearity of meropenem PK allows to calculate PTA for different doses based on the values obtained for the standard dosing (i.e. short-term infusion of 1 g of meropenem q8h). Basically, the PTA profiles corresponding to two different doses (i.e. 1000 and 2000 mg) are shifted by a constant equal to the difference in the logarithm of the two considered doses. PK parameters uncertainty was incorporated into the calculations of PTA using the parameters from the bootstrap samples.

## Results

A total of 20 patients were included in the study, yet the pharmacokinetic data was available from 19 patients to 5 females and 14 males, aged 36–79 years. One patient was excluded from the study due to complication during dialysis caused by dysfunction of cannula. For 9 patients samples were collected after the first dose of antibiotic, whereas for 10 patients during the consecutive days of antibiotic therapy. In the case of patients receiving meropenem at the moment of inclusion to the study, the samples were collected after the median time of 4 days (IQR 1–19) following initiation of the therapy. A summary of the patients’ characteristics is presented in Table 1.

**Table 1:** Patient characteristics of the study population

Parameter, unit	Median	Range	Number	%
Age, years	67	36–79	–	–
Body weight, kg	80	60–100	–	–
Gender				
Female	–	–	5	26.32
Male	–	–	14	73.68
Day of antibiotic therapy, days	2	1–12	–	–
Albumin concentration (ALB), g/l	24.6	15.6–31.8	–	–
Creatinine concentration, mg/dl	1.55	0.6–3.7	–	–
eGFR_MDRD_ (estimated with MDRD equation), ml/min	47	15–122	–	–
eGFR_CG_ (estimated with Cockroft–Gault equation), ml/min	50.29	17.5–134.8	–	–
Diuresis, ml/h	0	0–90	–	–
APACHE	31	8–44	–	–
SOFA	10	4–17	–	–
Presence or absence of sepsis according to surviving sepsis compaigne				
Septic	–	–	10	52.63
Nonseptic	–	–	9	47.37
CRRT				
CVVH and heparyn anticoagulation	–	–	9	47.37
CVVHD and citrate anticoagulation	–	–	10	52.63
Day of filter usage, days	1	1–3	–	–
Blood flow, ml/min	160	110–240	–	–
Dialysate/substitute flow, ml/h	2800	2100–3500	–	–
UF net, l/h	100	0–350	–	–

**Fig. 1:**
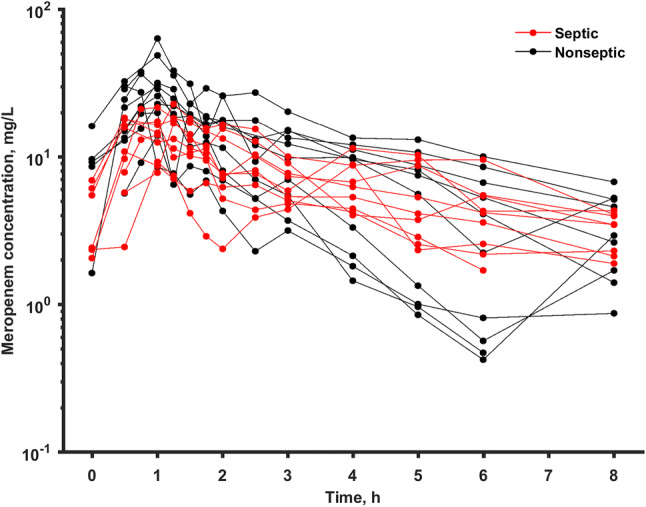
Raw concentration data stratified with respect to the presence of sepsis

The analyzed data consisted of 256 observations of meropenem concentration obtained from 19 patients. The raw concentration data is shown in Fig. 1. Two measurements from patients who did not receive the antibiotic before but had a positive plasma concentration at time 0 were treated as outliers and excluded from the analysis. A two-compartment model was used to describe the available data. The fits based one-compartment model were substantially worse (OFV was higher by 75.8). Typical PK parameters in population and inter-individual variability were estimated for *V*_1_, *V*_2_, *Cl*_CRRT_ and *Q.* The IIV for *Q* was fixed as it tended to zero. One significant covariate relationship between albumin concentration and *V*_1_ was identified during the model building process (OFV decrease by 13.3):

8 $$ V_{1,i} = 27.9\left( {{\text{ALB}}_{i} /24.6} \right)^{ - 2.8 7} \times \exp \left( {\eta_{{V_{1} ,i}} } \right). $$

**Fig. 2:**
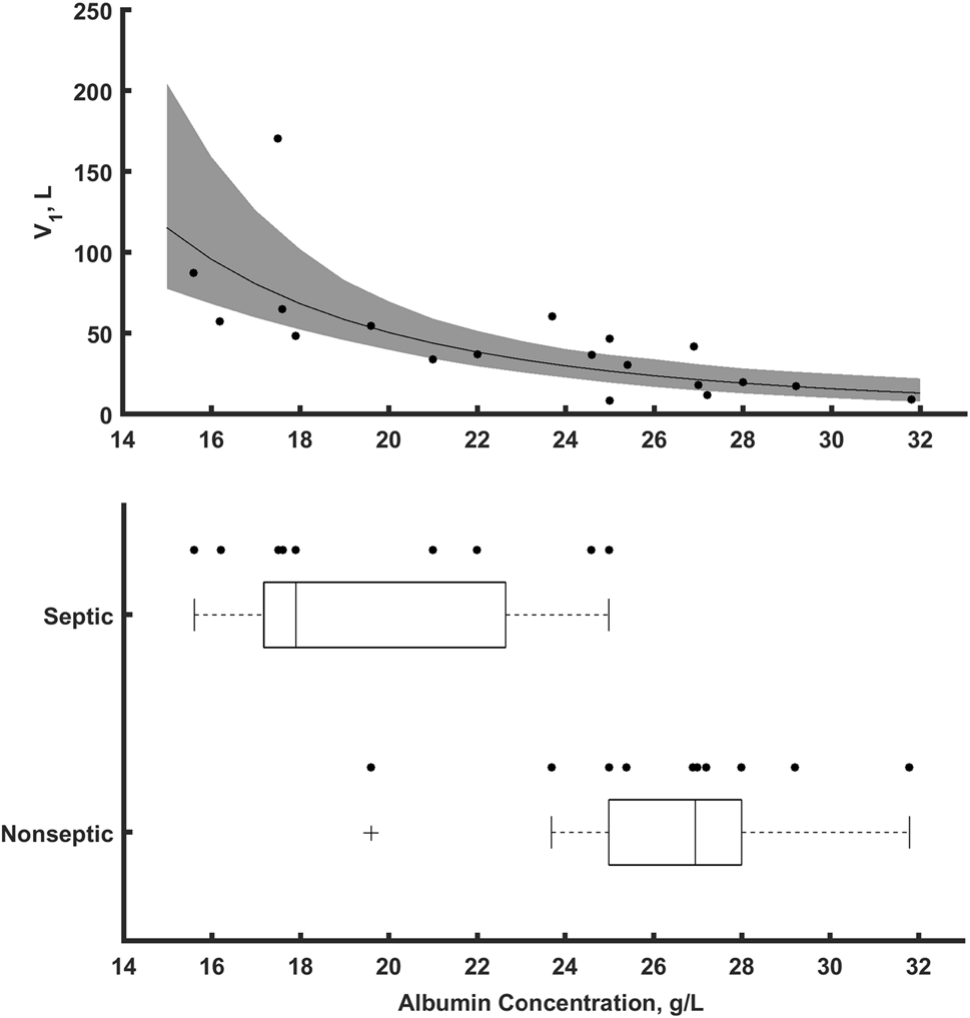
The upper graph shows the relationship between *V*_1_ and albumin concertation estimated during the covariate analysis along with individual values of PK parameters (points). The shaded areas correspond to 90% bootstrap-based uncertainty intervals for the line. The bottom graph shows the relationship between albumin concentration and the presence or absence of sepsis

The inclusion of this covariate into the model reduced considerably the estimated inter-individual variability for *V*_1_ from 82.3 to 53.1%. The identified covariate relationship is graphically presented in Fig. 2.

**Fig. 3:**
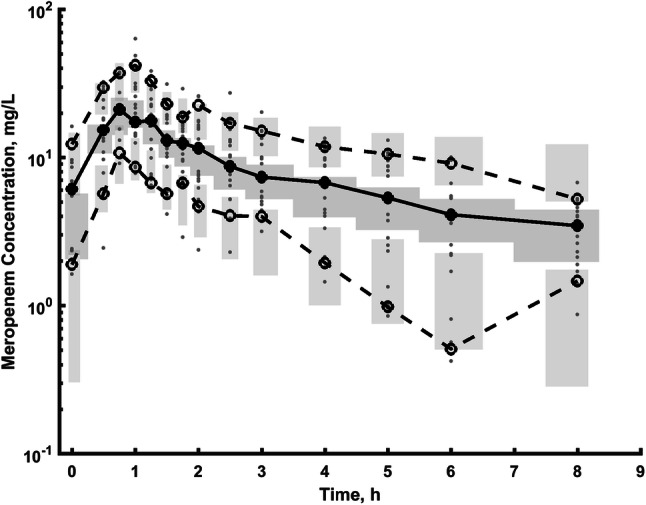
The visual predictive checks (VPC) plot shows the simulation-based 90% confidence intervals around the 5th, 50th, and 95th percentiles of the PK data in the form of a darker blue (50th) and light blue (5thand 95th) areas. The corresponding percentiles from the observed data are plotted in black color

Table 2 shows parameter estimates of the final population PK model of meropenem along with their bootstrap estimates. All PK parameters, inter-subject and residual error variances were estimated well with low relative standard error (RSE). The model parameters closely match the median estimates obtained from the bootstrap sample, which proves the final model is unbiased. The comparison of the mean PK parameter values obtained in this study with literature values is presented in Table 3. Typical GOF plots of the final model are presented in Supplemental Fig. 1S. The individual and population prediction versus observed concentrations are relatively symmetrically distributed around the line of identity. The conditional weighted residuals versus time and versus population predicted concentrations do not show any trend and are relatively evenly distributed around the zero. The VPC plot presented in Fig. 3 indicates that simulated data coincide with observed data. The individual predicted concentration versus time profiles were very close to the experimental data as presented in Supplemental Fig. 2S.

**Table 2:** Final model parameter estimates, 90% confidence interval of the parameter estimate derived from a nonparametric bootstrap analysis (*n* = 1000, unsuccessful = 8)

Parameter, units	Estimate	RSE (%)	Shrinkage (%)	Bootstrap median	Bootstrap 90% confidence interval
Fixed effects, *θ*					
$\theta_{{V_{1} }}$, l	27.9	17.9	–	28.5	20.8 to 37.8
$\theta_{{{\text{beta}},V_{1} }}$ (power function)	− 2.87	21.4	–	− 2.84	− 4.06 to − 1.80
$\theta_{{{\text{Cl }}_{{{\text{CRRT}}}} }}$, l/h	15.1	10.1	–	15.0	12.3 to 17.8
$\theta_{Q}$, l/h	21.1	16.4	–	21.1	13.1 to 26.9
$\theta_{{V_{2} }}$, l	33.7	28.1	–	34.6	20.0 to 78.0
Inter-individual variability, $\omega_{P}^{2}$					
$\omega_{{V_{1} }}^{2}$, %CV	53.1	23.0	8.1	47.6	28.3 to 47.6
$\omega_{{{\text{Cl}}}}^{2}$, %CV	43.7	12.5	1.1	42.0	31.0 to 52.2
$\omega_{Q}^{2}$, %CV	0 fixed	–	–	–	–
$\omega_{{V_{2} }}^{2}$, %CV	85.6	25.5	30.1	82.9	0.10 to 138
Residual error model, *σ*^2^					
*σ*_add,_ mg/l	0.881	28.4	–	0.874	0.208 to 1.50
$\sigma_{{{\text{prop}}}}^{2}$_,_ %CV	24.1	10.5	–	23.5	18.3 to 28.6

RSE denotes residual standard error; %CV = sqrt(exp(IIV^2^ − 1)·100% and IIV denoted inter-individual variability (variance)

**Table 3:** Comparison of the results of current study with the literature data

Author	Description of the model	PK parameters				
		*V*_1_ (l)	*V*_2_ (l)	*V*_ss_ (l)	Cl (l/h)	*Q*_2_ (l/h)
Current study	Two-compartment model (critically ill patients during CRRT)	27.9 (80 kg patient, albumin concentration of 24.6 g/l)	33.7	61.6 (80 kg patient, albumin concentration of 24.6 g/l)	15.1	21.1
Jaruratanasirikul [14]	One-compartment model (healthy volunteers)	–	–	11.94	12.97	–
Jaruratanasirikul [15]	One-compartment model (patients with sepsis or septic shock treated in ICUs)	–	–	23.7	11.4 (Patient with eGFR of 120 ml/min)	–
Ulldemolins [16]	One-compartment model (patients with septic shock during renal replacement therapy)	–	–	30.2 (70 kg patient)	8.1 (Patient with daily diuresis of 2000 ml)	–
Chung [17]	Two-compartment model	14.3	17.7	32.0	11.7 (Patient with eGFR of 120 ml/min)	15.9
Roberts [18]	Two-compartment model (patients with sepsis)	7.9	14.8	22.7	16.3 (Patient with eGFR of 120 ml/min)	56.3
Ehmann [24]	Two-compartment model (critically ill patients without CRRT)	7.89	16.1	24.0	9.25 (Patient with eGFR_CG_ of 80.8 ml/min)	28.4

**Fig. 4:**
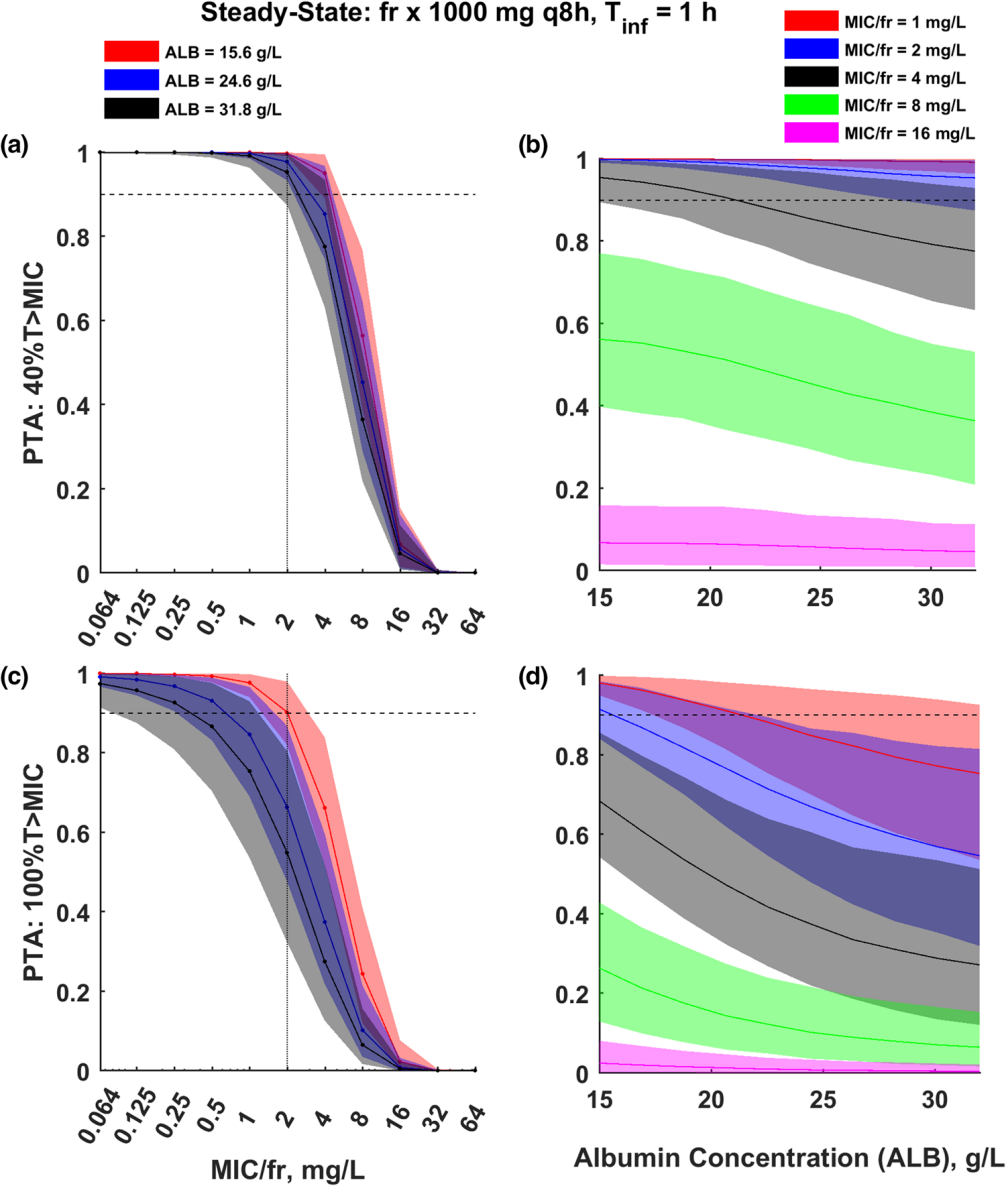
PTA of %*T* > MIC versus **a**, **c** MIC and **b**, **d** albumin concertation during the steady-state conditions observed after multiple dosing of meropenem at a 1000 mg q8h given as an 1 h infusion. The plasma concentration maintenance above MIC for **a**, **b** 40% and **c**, **d** 100% of the time during 24 h period was used as a target. The horizontal line denotes PTA of 90%. Colored dots, lines and shaded areas correspond to median and 90% CI of the PTA (bootstrap-based uncertainty intervals). *fr* (equal to Dose/1000) allows to calculate the PTA profile for different dose, i.e. *fr* = 2 corresponds to the dose of 2000 mg

Simulations of the model were used to assess the impact of albumin on the PTA (40%*T* > MIC and 100%*T* > MIC) for 1-h infusion of 1000 mg q8h and steady-state conditions. The PTA versus MIC and albumin concentrations profiles are shown in Fig. 4. PTA analysis showed an increasing PTA for patients with lower albumin concentrations. The PTA values (CI) of 40%*T* > 2 mg/l target were 99.8% (99.0–100), 97.8 (93.5–99.9) and 95.3 (87.4–9.7) for albumin concentration of 15.6, 24.6 and 31.8 g/l. The PTA values (CI) for 100%*T* > 2 mg/l target observed in this study were 91.8% (84.4–98.4), 70.0 (51.6–88.4) and 58.7 (36.2–83.1) for albumin concentration of 15.6, 24.6 and 31.8 g/l (Fig. 4). It indicates that a larger dose is needed to achieve this more stringent pharmacological target. 100%*T* > MIC target is achieved by 90% of patients with albumin of 15.6, 24.6 and 31.8 g/dl for MIC = 2.0 (CI 1.3–3.2), 0.69 (CI 0.26–1.7), 0.37 (CI 0.09–1.2). Thus, about onefold (CI 0.6–1.5), 2.9-fold (CI 1.2–7.7) and 5.4-fold (CI 1.7–22) increase in dose is needed to achieve 90% PTA using 100%*T* > 2 mg/l target for patients with albumin of 15.6, 24.6 and 31.8 mg/dl. Please note that there is large uncertainty in these predictions, that should be taken into account during the decision making. The PTA versus MIC and versus albumin concentrations profiles for 1000 mg q8h and infusion duration of 30 min and 3 h, and for 1000 mg q12h and infusion duration of 30 min, 1 h and 3 h are shown in Supplemental Figs. 3S–7S. These graphs suggest slightly higher PTA values for longer infusion durations and lower PTA values for 1000 mg q12h dosing regimen than for 1000 mg q8h. The provided figures also allow to obtain PTA values for different doses using *fr* (equal to the Dose/1000) as a conversion factor. This conversion factor implies that exactly the same PTA values are expected for 1000 mg q8h and %*T* > 2 mg/l target as for 2000 mg q8h and %*T* > 4 mg/l target.

## Discussion

The presence of AKI and the impact of extracorporeal therapies on the ability of antibiotics to achieve therapeutic concentrations remains one of the greatest challenges for clinicians prescribing antimicrobials in the ICU [14]. Since recommended beta-lactam regimens are often inadequate in septic patients treated with CRRT, the drug concentrations might be too low to ensure adequate bacterial killing, resulting in increased morbidity and mortality, as well as the emergence of antibiotic resistance. Despite numerous studies on meropenem pharmacokinetics in the critically ill patients published to date, there is no consensus on dosing of this widely used antimicrobial in different ICU scenarios [4, 15–20]. In this observational single-center cohort study, performed at a tertiary mixed ICU, we aimed to characterize the sources of PK variability of meropenem in a diverse population of critically ill patients receiving CRRT and to perform dosing simulations to assess their probability of target attainment (PTA), in order to provide empirical dosing recommendations.

The population PK model was successfully developed to describe the time course of meropenem concentrations in critically ill patients undergoing CRRT. The inclusion of albumin resulted in almost 30% decrease of inter-individual variability for *V*_1_ indicating its clinical significance for dosing decision that are influenced by this parameter. Albumin concentration was selected in the final model as a parameter reflecting the severity of sepsis. Please note that there is a clear relationship between albumin concentration and the presence of sepsis among patients. Septic patients had lower albumin concentration compared to non-septic patients (Fig. 3). The majority of the mean PK parameter estimates obtained in our study were in agreement with the ones obtained by other authors and are presented in Table 3 [4, 15–20]. The typical clearance values are similar, yet the volume of distribution at steady state (*V*ss = *V*_1_ + *V*_2_) is not consistent between the studies. Clearly, the *V*_ss_ is higher than values from previous studies in adults with sepsis and CRRT [15, 16]. Only for high albumin concentrations (non-septic patients) *V*_ss_ tends to values similar to values reported in the literature (33.7 l). The possible reason for such discrepancies might be due to the study difference, i.e. difference in the health status of the patients (sepsis severity).

Meropenem is a hydrophilic, small molecule antibiotic, with a relatively low volume of distribution and a very low level of protein binding (2%). The abovementioned features enable easy removal of the drug by the kidneys, as well as during CRRT [21]. Therefore, it could be anticipated that both patients’ diuresis and CRRT intensity should be significant modifiers of meropenem clearance. Interestingly, in this work we were unable to show the influence of the CRRT intensity on the inter-individual variability of PK parameters (especially *Cl*_CRRT_), which is in line with the results obtained in a study performed on similar patient population [17]. In contrast to the beforementioned study, we did not observe residual diuresis to significantly alter meropenem PK. On the other hand, contrary to our results Ulldemolins and colleagues [17] did not identify albumin levels to modify meropenem volume of distribution. Hypoalbuminaemia is likely to increase the total volume of distribution and clearance of many widely used antimicrobials. This phenomenon might translate to lower drug exposures, resulting in failing to attain the pharmacodynamic targets, especially for time-dependent antibiotics. In a comprehensive review on the effects of hypoalbuminemia on optimizing antibacterial dosing in critically ill patients, the authors emphasized the possible impact of sepsis-driven alterations in the degree of protein binding of many highly protein-bound antibacterials, which could lead to altered pharmacokinetics and pharmacodynamics [22]. Meropenem belongs to the group of minimally bound antimicrobials, therefore the possible impact of hypoalbuminemia on its pharmacokinetics due to alternation of drug binding is unlikely. Since for the studied patients, the hypoalbuminemia is associated with the presence of sepsis, the increased volume of distribution in patients with sepsis (with low albumin concertation) might be caused by other sepsis-related mechanism, like the increased capillary permeability. The increased capillary permeability would affect both the concentration of albumin and volume of distribution of meropenem. In such a case the association between them could be explained by a sepsis being a common cause affecting the albumin concentration and volume of distribution of meropenem. Nevertheless, to fully assess the exact mechanism a more detailed casual model is required.

Simulations of the model allowed us to assess the impact of albumin concentration on PTA for the applied standard dosing regimen. %*T* > MIC seems to be the best parameter that correlates with the bactericidal activity of meropenem [15]. Traditionally used PK/PD target for meropenem requires maintenance of blood antibiotic concentration above MIC for 40% of the dosing interval, yet it might be insufficient for critically ill patients. A more aggressive target of meropenem concentration exceeding MIC for 100% of dosing interval was proposed mainly due to many pathophysiological changes which may influence the drug pharmacokinetics in the critically ill patients. It is noteworthy that even higher targets such as *T* > 4 × MIC or even *T* > 5 × MIC are advocated by some researchers [7, 23]. To achieve the traditional target of 40%*T* > MIC, standard dosage of 1 g every 8 h is sufficient for empirical treatment of the most common pathogens with MIC < 2 mg/l [24]. It is also sufficient for the studied patients (Fig. 4). However, to achieve more aggressive target of 100%*T* > MIC, especially in patents with high albumin concentrations, standard dosage might be insufficient. Even lower PTA may be anticipated when targeting the %*T* > 4–5 × MIC. In our model, hypoalbuminemia was associated with a greater possibility of PTA for considered dosing schemes (Figs. 4, 3S–7S). The possible explanation of the observed phenomenon includes the impact of significantly larger total volume of distribution associated with hypoalbuminemia on the terminal half time of meropenem, which might be advantageous for sustaining sufficient antimicrobial concentrations throughout the entire dosing interval to ensure effective bacterial killing.

The main limitation of the study includes relatively small sample size, large uncertainty intervals, as well as a significant population diversity, typical for a mixed ICU environment. Another important limitation was not measuring meropenem concentrations in the ultrafiltrate, leading to lack of estimation of sieving coefficient, which could allow us to more accurately calculate the total clearance of the drug during CRRT. To date, several studies were published on the possible adsorption of meropenem on different types of membranes used during CRRT. Nevertheless, despite demonstrating that meropenem appears to be rapidly absorbed into the CRRT circuit when polysulfon membranes are used, these findings are likely to be clinically insignificant and not affect dosing requirements [25]. The strength of this study is that it included septic and non-septic patients studied under the same methodology. It allowed us to directly compare the difference in PK between the two group of patients. In addition, the identified covariate relationship provides a means to adjust the dose to obtain comparable PTA for septic and non-septic patients undergoing CRRT.

## Conclusions

The population PK model was successfully developed to describe the time course of meropenem concentrations. The inclusion of albumin as a covariate resulted in almost 30% decrease of inter-individual variability for *V*_1_. It is particularly important as the attainment of the pharmacodynamics index for meropenem is sensitive to the value of the volume of distribution and clearance. Simulations of the model allowed to assess the impact of albumin concentration on PTA of %*T* > MIC. The hypoalbuminemia was found to be associated with higher PTA in the CRRT patients at steady-state conditions after multiple meropenem administrations every 8 h as an 1 h infusions.

## Electronic supplementary material

Below is the link to the electronic supplementary material.

Supplementary file1 (DOCX 2452 kb)
