# Supplementary material for: Population pharmacokinetics of standard-dose meropenem in critically ill patients on continuous renal replacement therapy: a prospective observational trial
Source: Pharmacol Rep. 2020 Apr 16;72(3):719–29. doi: 10.1007/s43440-020-00104-3 (PMC7329797; doi:10.1007/s43440-020-00104-3)
Supplement: Supplementary file 1 — Supplementary file1 (DOCX 2452 kb) [file 43440_2020_104_MOESM1_ESM.docx]

**SUPPLEMENTARY ONLINE MATERIAL FOR**

Population pharmacokinetics of standard dose meropenem in critically ill patients on continuous renal replacement therapy – a prospective observational trial.

Dariusz Onichimowski D, Anita Będźkowska, Hubert Ziołkowski, Jerzy Jaroszewski, Michał Borys, Mirosław Czuczwar, Paweł Wiczling


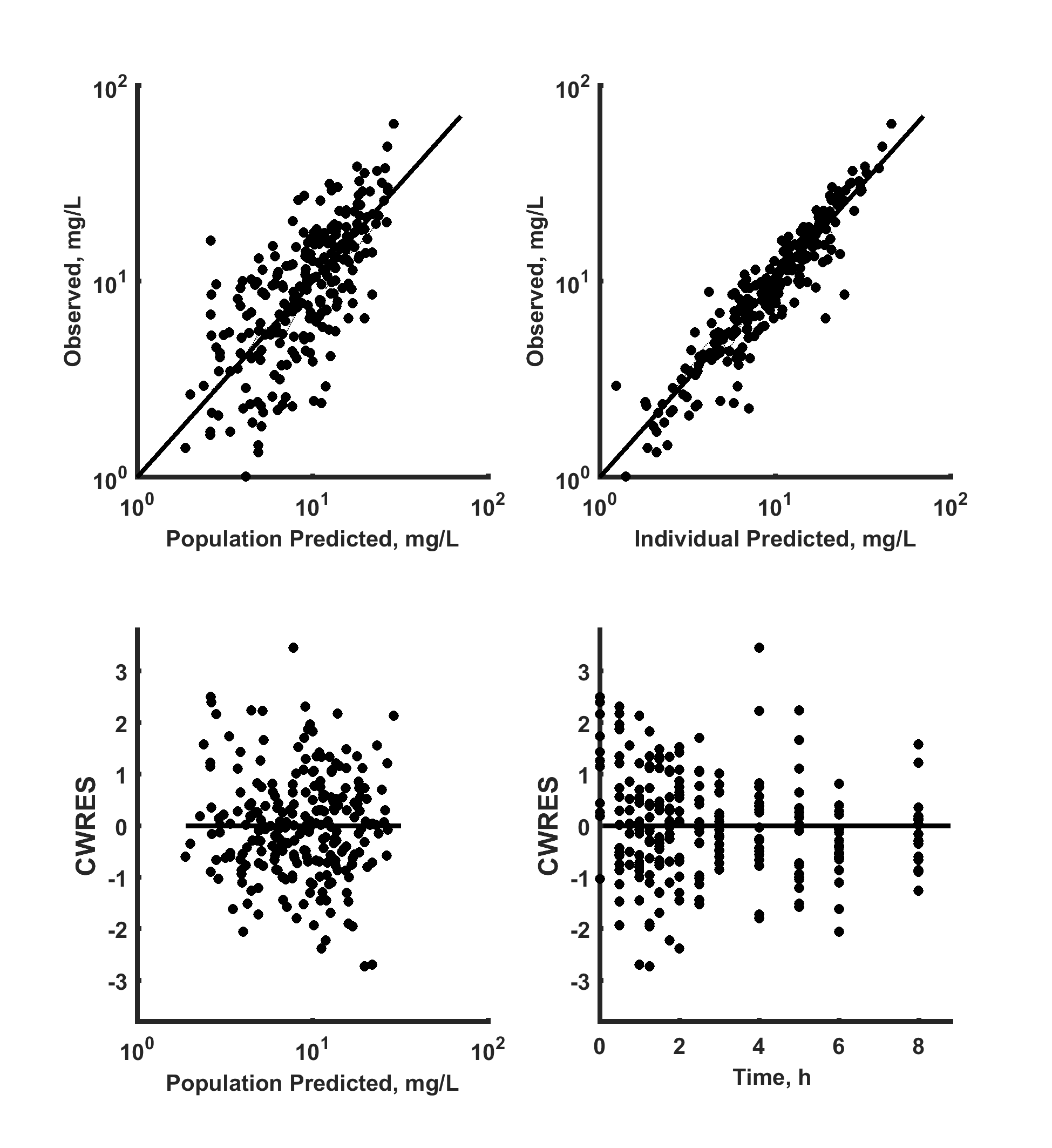


Figure 1S. Goodness of fit plots: the observed versus the population predicted concentrations; the observed versus the individual population predicted concentrations; conditional weighted residuals (CWRES) versus population predicted concentrations and time.


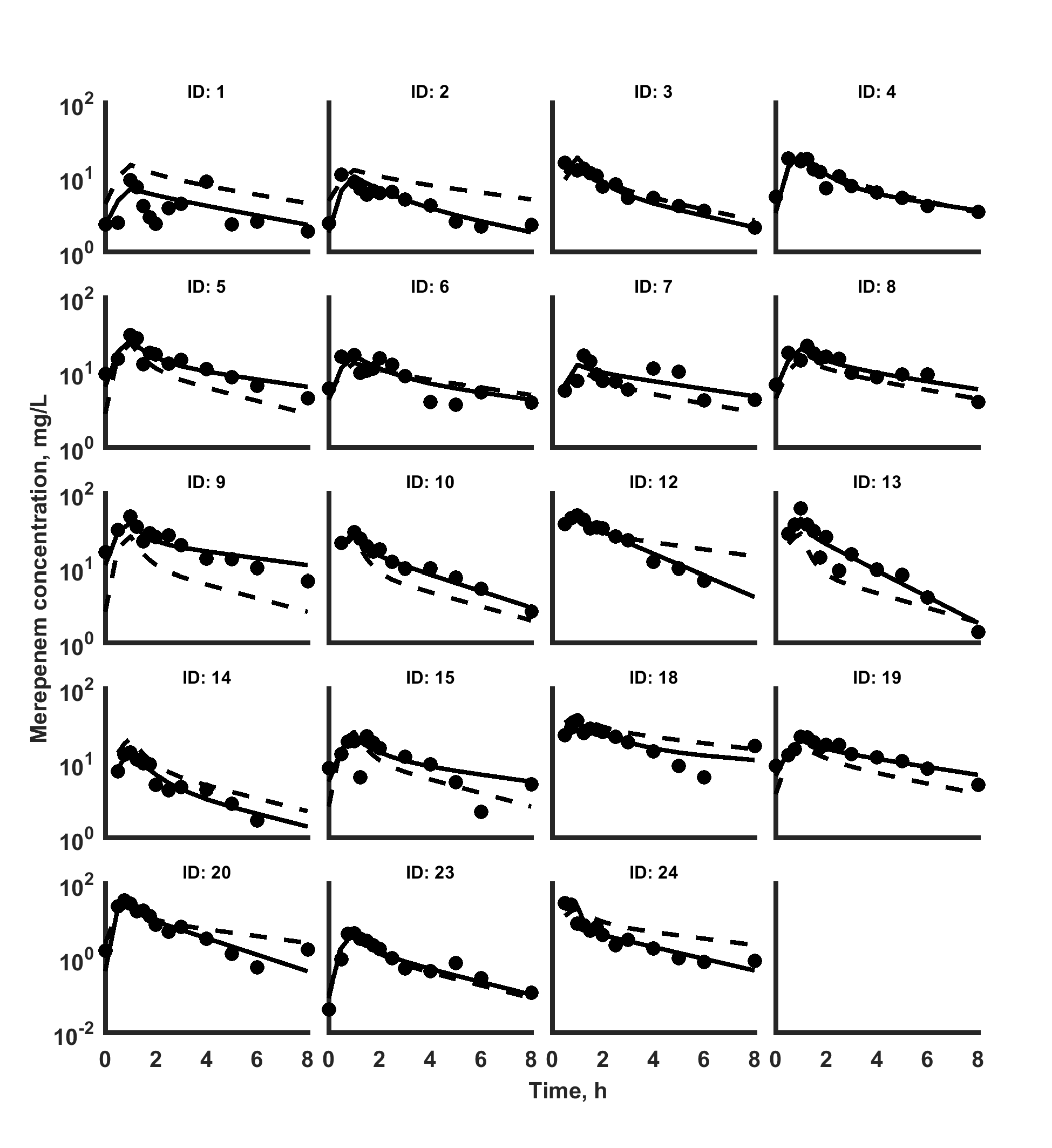


Figure 2S. Experimental (points), individual (solid lines) and population (dashed lines) model predictions of meropenem concentrations.


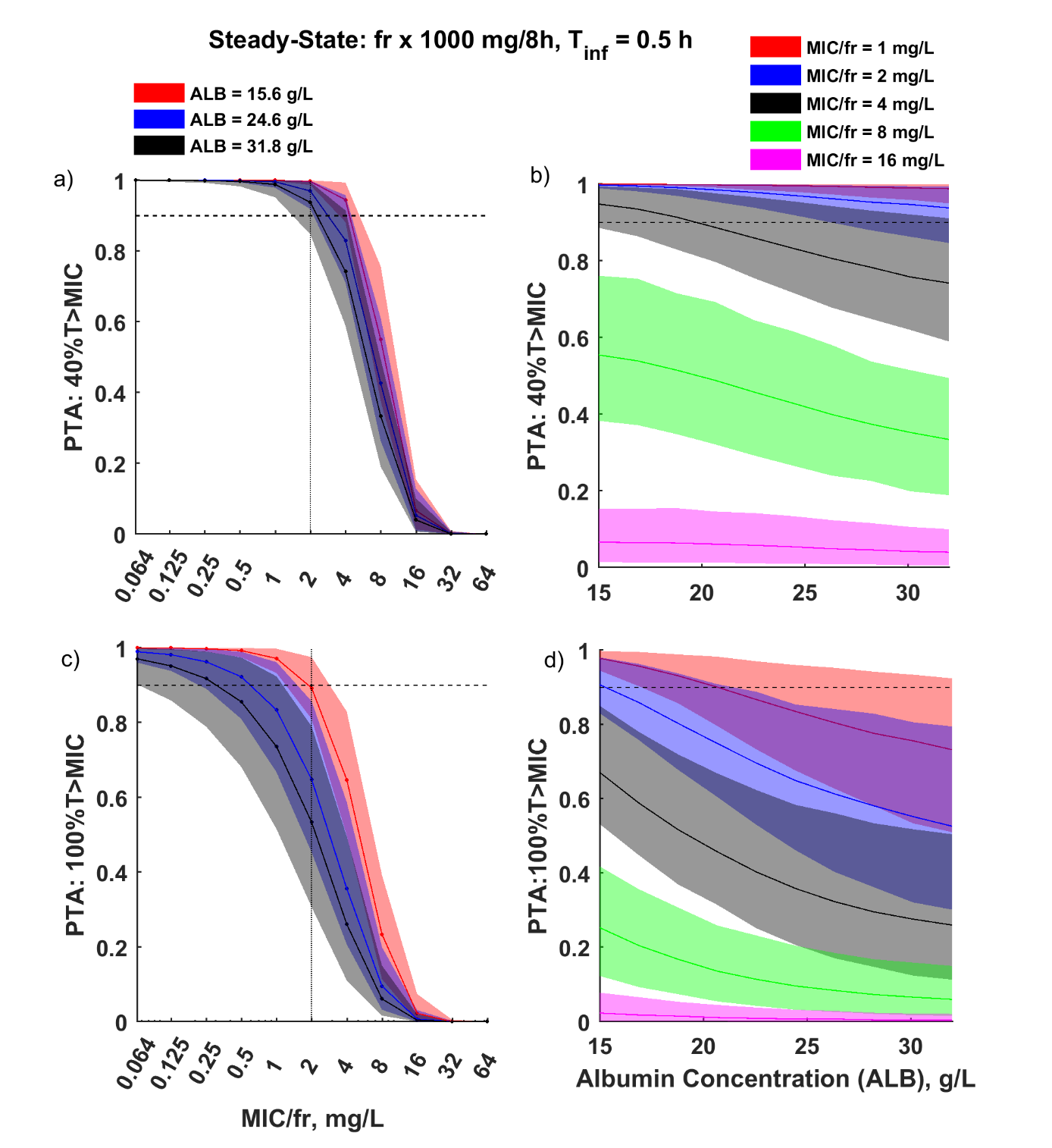


Figure 3S. PTA of %T>MIC versus (a and c) MIC and (b and d) albumin concertation during the steady-state conditions observed after multiple dosing of meropenem at a 1000 mg q8h given as an 30 min infusion. The plasma concentration maintenance above MIC for (a and b) 40% and (c and d) 100% of the time during 24 h period was used as a target. The horizontal line denotes PTA of 90%. Colored dots, lines and shaded areas corresponds to median and 90% CI of the PTA (bootstrap-based uncertainty intervals). *fr* = Dose/1000 allows to calculate the PTA profile for different dose, i.e. *fr* = 2 corresponds to the dose of 2000 mg.


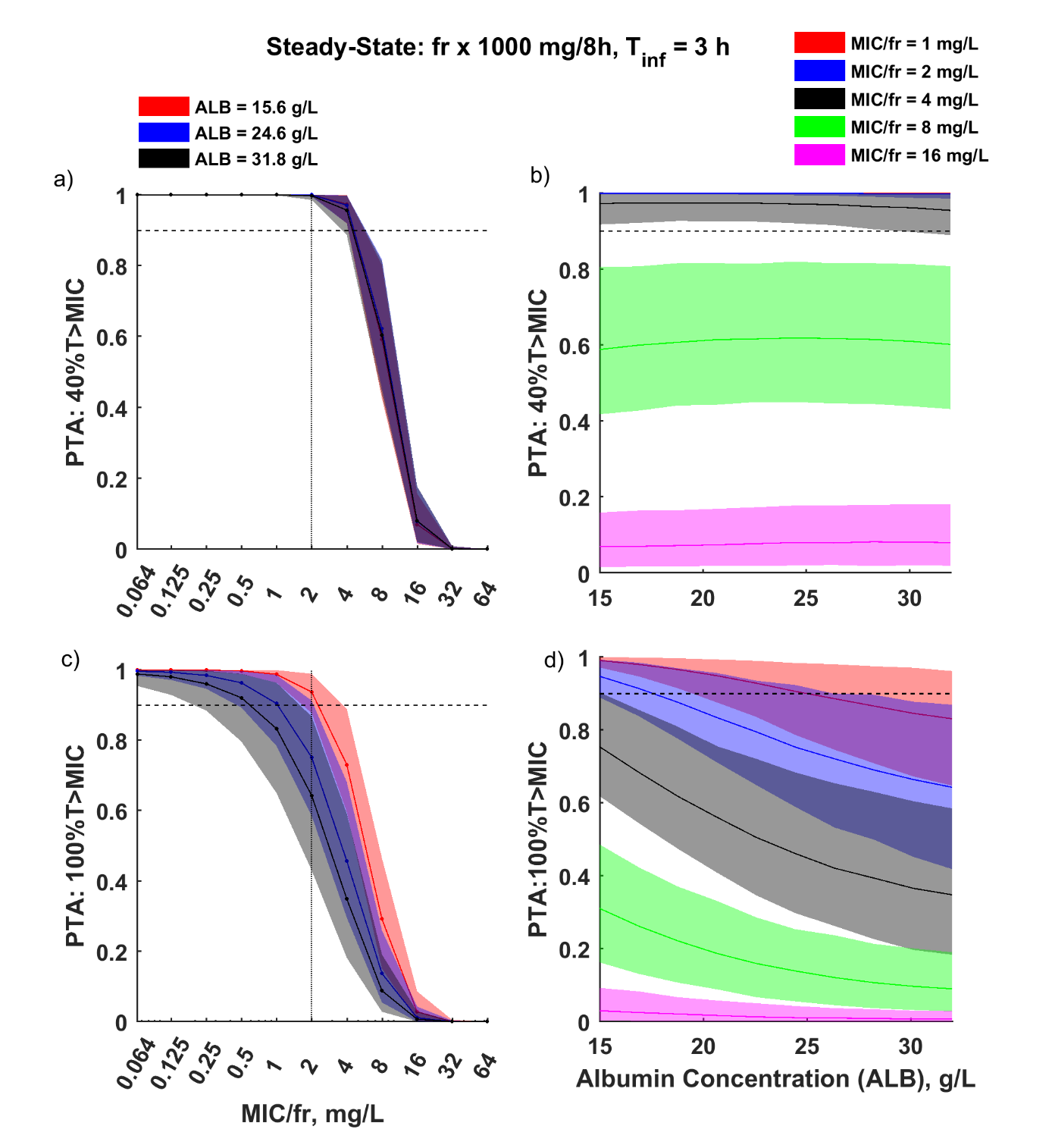


Figure 4S. PTA of %T>MIC versus (a and c) MIC and (b and d) albumin concertation during the steady-state conditions observed after multiple dosing of meropenem at a 1000 mg q8h given as a 3 h infusion. The plasma concentration maintenance above MIC for (a and b) 40% and (c and d) 100% of the time during 24 h period was used as a target. The horizontal line denotes PTA of 90%. Colored dots, lines and shaded areas corresponds to median and 90% CI of the PTA (bootstrap-based uncertainty intervals). *fr* = Dose/1000 allows to calculate the PTA profile for different dose, i.e. *fr* = 2 corresponds to the dose of 2000 mg.


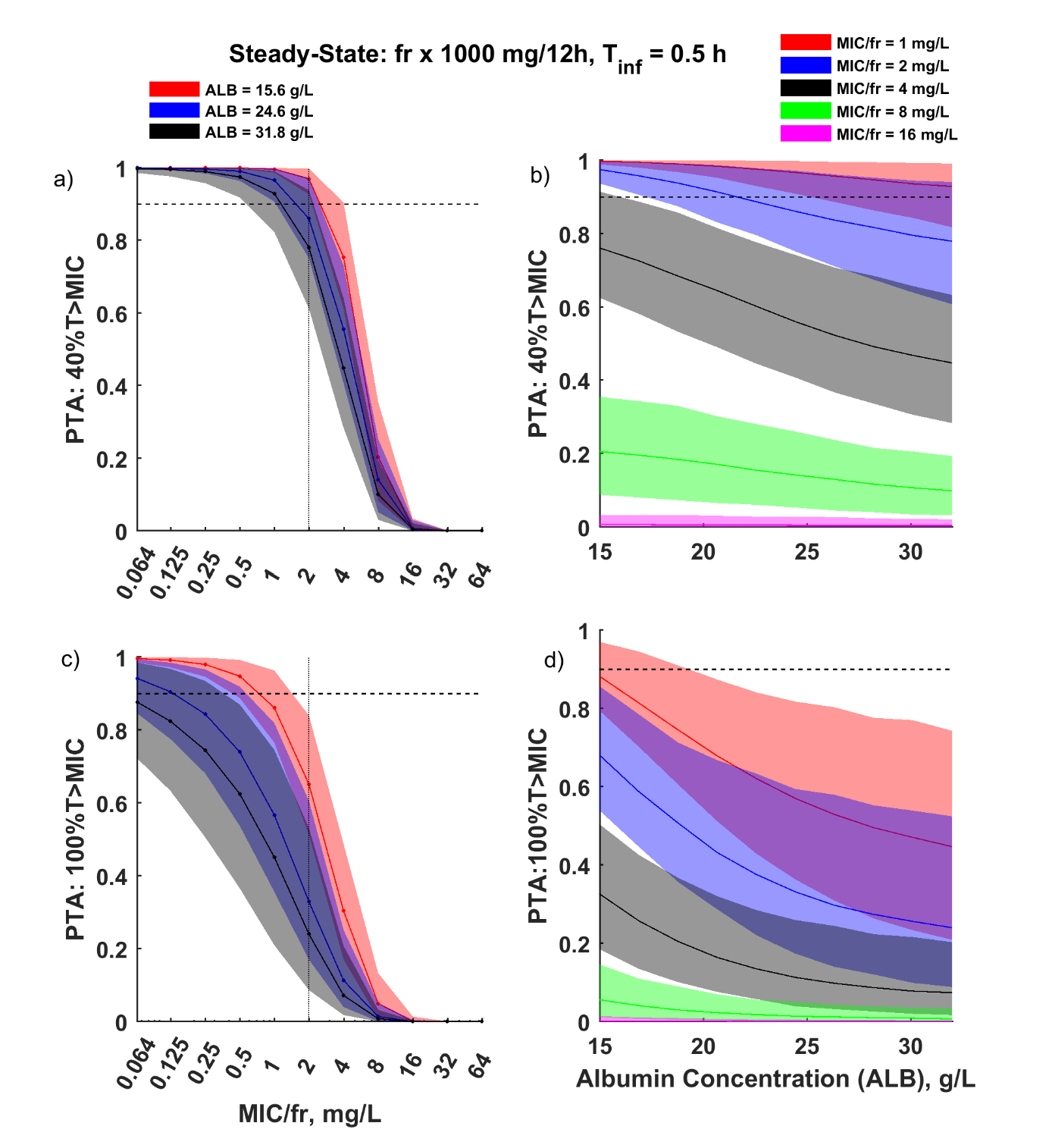


Figure 5S. PTA of %T>MIC versus (a and c) MIC and (b and d) albumin concertation during the steady-state conditions observed after multiple dosing of meropenem at a 1000 mg q12h given as a 30 min infusion. The plasma concentration maintenance above MIC for (a and b) 40% and (c and d) 100% of the time during 24 h period was used as a target. The horizontal line denotes PTA of 90%. Colored dots, lines and shaded areas corresponds to median and 90% CI of the PTA (bootstrap-based uncertainty intervals). *fr* = Dose/1000 allows to calculate the PTA profile for different dose, i.e. *fr* = 2 corresponds to the dose of 2000 mg.


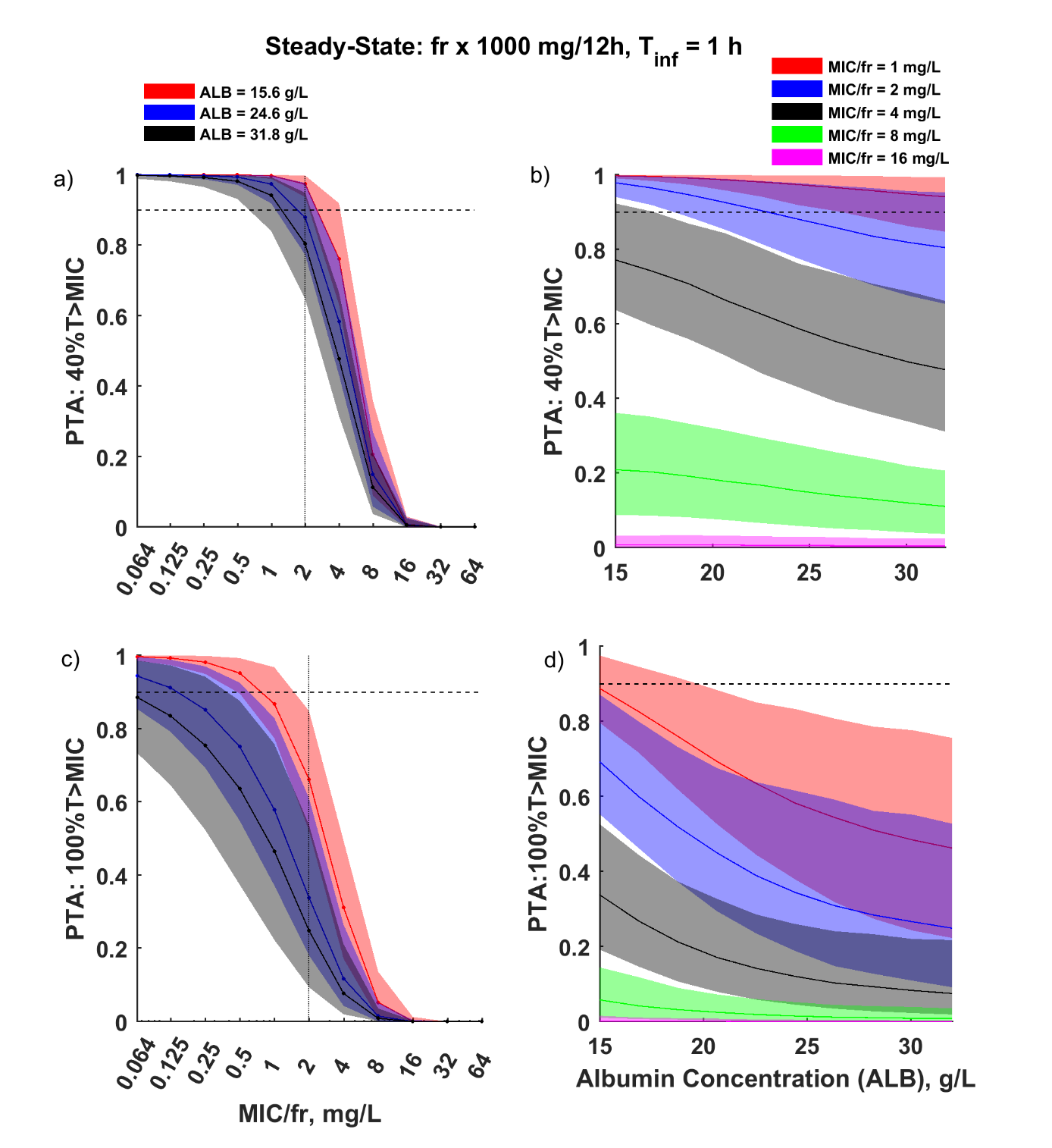


Figure 6S. PTA of %T>MIC versus (a and c) MIC and (b and d) albumin concertation during the steady-state conditions observed after multiple dosing of meropenem at a 1000 mg q12h given as a 1 h infusion. The plasma concentration maintenance above MIC for (a and b) 40% and (c and d) 100% of the time during 24 h period was used as a target. The horizontal line denotes PTA of 90%. Colored dots, lines and shaded areas corresponds to median and 90% CI of the PTA (bootstrap-based uncertainty intervals). *fr* = Dose/1000 allows to calculate the PTA profile for different dose, i.e. *fr* = 2 corresponds to the dose of 2000 mg.


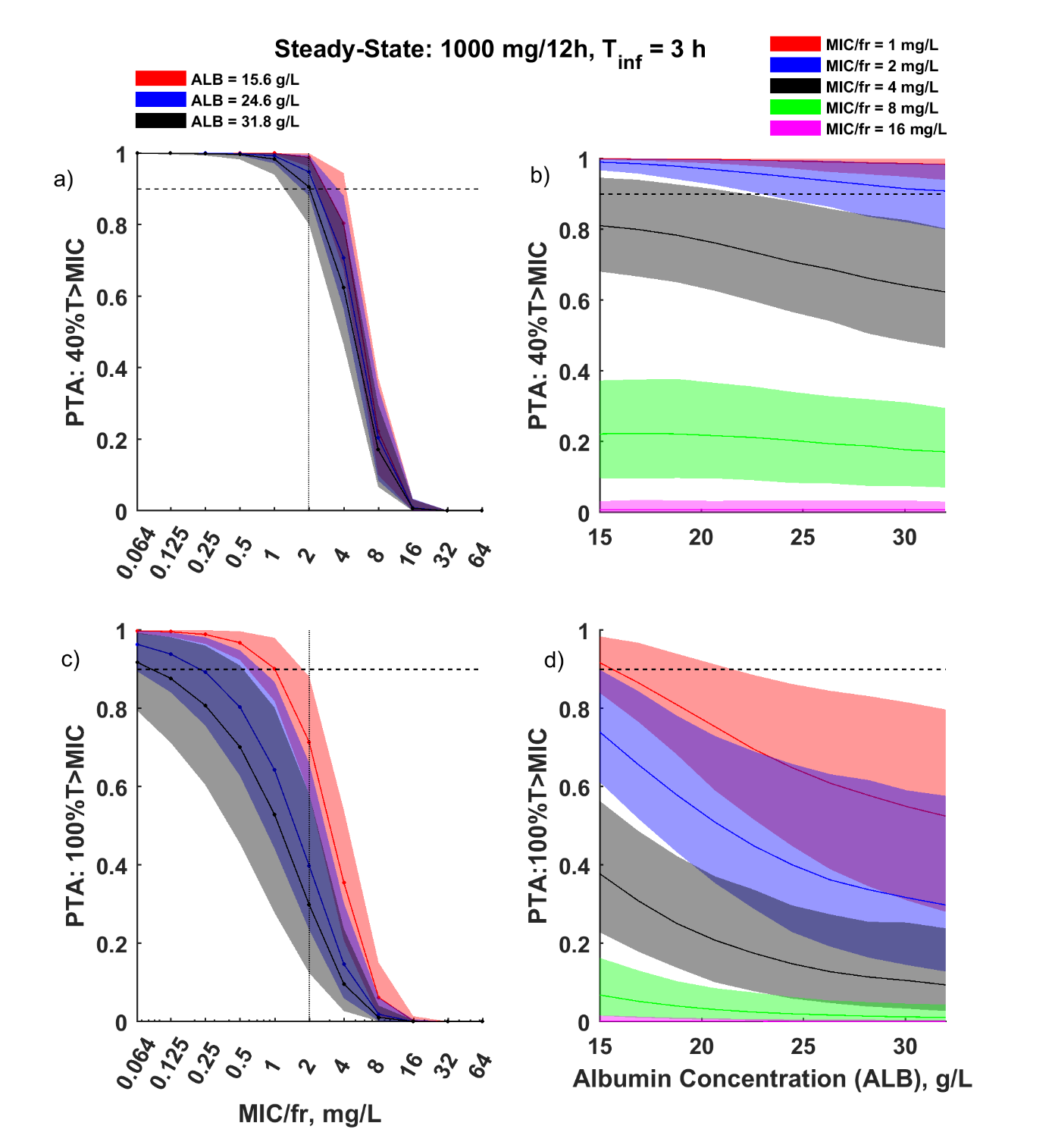


Figure 7S. PTA of %T>MIC versus (a and c) MIC and (b and d) albumin concertation during the steady-state conditions observed after multiple dosing of meropenem at a 1000 mg q12h given as a 3 h infusion. The plasma concentration maintenance above MIC for (a and b) 40% and (c and d) 100% of the time during 24 h period was used as a target. The horizontal line denotes PTA of 90%. Colored dots, lines and shaded areas corresponds to median and 90% CI of the PTA (bootstrap-based uncertainty intervals). *fr* = Dose/1000 allows to calculate the PTA profile for different dose, i.e. *fr* = 2 corresponds to the dose of 2000 mg.
